# High-Fat Feeding Impairs Nutrient Sensing and Gut Brain Integration in the Caudomedial Nucleus of the Solitary Tract in Mice

**DOI:** 10.1371/journal.pone.0118888

**Published:** 2015-03-16

**Authors:** Althea R. Cavanaugh, Gary J. Schwartz, Clémence Blouet

**Affiliations:** 1 Department of Medicine of The Albert Einstein College of Medicine of Yeshiva University, Bronx, New York, United States of America; 2 Institute of Metabolic Science, Medical Research Council Metabolic Disease Unit, University of Cambridge, Cambridge, United Kingdom; INRA, FRANCE

## Abstract

Hyperphagic obesity is characterized in part by a specific increase in meal size that contributes to increased daily energy intake, but the mechanisms underlying impaired activity of meal size regulatory circuits, particularly those converging at the caudomedial nucleus of the solitary tract in the hindbrain (cmNTS), remain poorly understood. In this paper, we assessed the consequences of high-fat (HF) feeding and diet-induced obesity (DIO) on cmNTS nutrient sensing and metabolic integration in the control of meal size. Mice maintained on a standard chow diet, low-fat (LF) diet or HF diet for 2 weeks or 6 months were implanted with a bilateral brain cannula targeting the cmNTS. Feeding behavior was assessed using behavioral chambers and meal-pattern analysis following cmNTS L-leucine injections alone or together with ip CCK. Molecular mechanisms implicated in the feeding responses were assessed using western blot, immunofluorescence and pharmacological inhibition of the amino acid sensing mTORC1 pathway (mammalian target of rapamycin complex 1). We found that HF feeding blunts the anorectic consequences of cmNTS L-leucine administration. Increased baseline activity of the L-leucine sensor P70 S6 kinase 1 and impaired L-leucine-induced activation of this pathway in the cmNTS of HF-fed mice indicate that HF feeding is associated with an impairment in cmNTS mTOR nutritional and hormonal sensing. Interestingly, the acute orexigenic effect of the mTORC1 inhibitor rapamycin was preserved in HF-fed mice, supporting the assertion that HF-induced increase in baseline cmNTS mTORC1 activity underlies the defect in L-leucine sensing. Last, the synergistic feeding-suppressive effect of CCK and cmNTS L-leucine was abrogated in DIO mice. These results indicate that HF feeding leads to an impairment in cmNTS nutrient sensing and metabolic integration in the regulation of meal size.

## Introduction

Growing evidence indicates that distributed populations of specialized neurons in the brain can detect and integrate a variety of metabolic signals, and engage downstream circuits contributing to the feedback inhibitory regulation of food intake [1]. The caudomedial nucleus of the solitary tract (cmNTS) represents one of the key nodes of sensing and integration important to the regulation of feeding, particularly in the direct control of meal size [2; 3]. Localized at the interface between the periphery and higher brain structures, the cmNTS is uniquely positioned to integrate gustatory and visceral inputs with cortico-limbic inputs, and relay the integrated results to nearby medullary motor output circuits [4]. Increasing neuroanatomical and functional evidence supports the physiological relevance of this model [3; 5–7], and one of its most recent developments is the demonstration that the cmNTS also detects local levels of nutrients and integrates this information with gut-derived, hormonal and forebrain descending inputs in the control of feeding [8; 9].

Behavioral analyses of animals acutely or chronically maintained on high-energy diets indicate that hyperphagia in those conditions is driven at least in part by an increase in meal size, possibly explained by enhanced orosensory stimulation and/or reduced sensitivity to postoral inhibitory feedback, whereas the regulation of meal frequency is relatively preserved [10; 11]. Consistent with an alteration in the processing of postoral inhibitory signals regulating meal size, decreased responsiveness to intestinal lipids and exogenous gut peptides such as CCK or GLP-1 has been reported in rodents fed a HF diet [12; 13] [14]. Further supporting this notion, rodents fed a HF diet exhibit decreased intestinal expression of CCK- and GLP1-receptors, and decreased activation of hindbrain neurons in response to gut lipid infusion [15; 16]. Together, these data suggest a role for impaired gut-hindbrain satiation signaling in hyperphagic obesity. However, little is known about the possible alterations in the cmNTS responses to direct nutrient exposure, or in the cmNTS integrative capacity in HF-fed animals. Our previous work indicates that central sensing of postprandial leucine contributes to the negative feeding inhibition mechanisms implicated in the regulation of food intake [8; 17]. Consequently, in this study, we tested the hypothesis that HF feeding diminishes the anorectic effects of both direct cmNTS L-leucine sensing and the integration of cmNTS L-leucine with systemic CCK.

## Materials and Methods

### Animals

Experiments were performed on male C57BL/6 or POMC-EGFP mice (Jackson Laboratories, Stock number 009593) purchased from the Jackson Laboratories. Mice were obtained between 8 and 10 weeks of age and maintained on chow, HF (D12266B, 31.8%k kcal fat, 4.4 kcal/g) or a control low fat diet (LF, D12489B, 4.6%fat, 3.8 kcal/g) (Research Diets). All animals were housed in individual cages and maintained in a temperature-controlled room under a standard 12h/12h light/dark cycle with *ad libitum* access to water and food. Before any brain injection, animals were adapted to the brain injection system for at least 4 consecutive days preceding the injection. All experimental protocols were approved by the Institute for Animal Studies of the Albert Einstein College of Medicine.

### CNS surgery

Surgical procedures were performed under ketamine/xylazine anesthesia. Animals were stereotaxically implanted with bilateral steel guide cannulae (Plastics One) positioned 2mm above the caudomedial nucleus of the solitary tract (cannula holding bar in a 10° rostro-caudal angle, coordinates relative to occipital suture: A/P +0.5 mm, D/V-3 mm. +/- 0.4 lateral to midline). Beveled stainless steel injectors (33 gauge mounted onto a 26-gauge sleeve) extending 2.0 mm from the tip of the guide cannulas were used for injections. Animals were allowed a 1-week recovery. Correct bilateral NTS cannula placement was confirmed histologically postmortem.

### Feeding behavior studies and injection designs

Age-matched animals were adapted for 1 week to individual feeding chambers (Med Associates) with *ad libitum* access to LF or HF pellets (Bioserv: 20 mg precision pellets, F05524 and F06294). Meal patterns were determined using the following criteria: a meal was described as the removal of five or more pellets (total mass of 0.100 g), with a maximum inter-pellet interval of 5 min. Injections were performed in a crossover manner according to the injection designs described below, and at least 3 days elapsed between each injection.

#### Injection design 1

**Fig 1:**
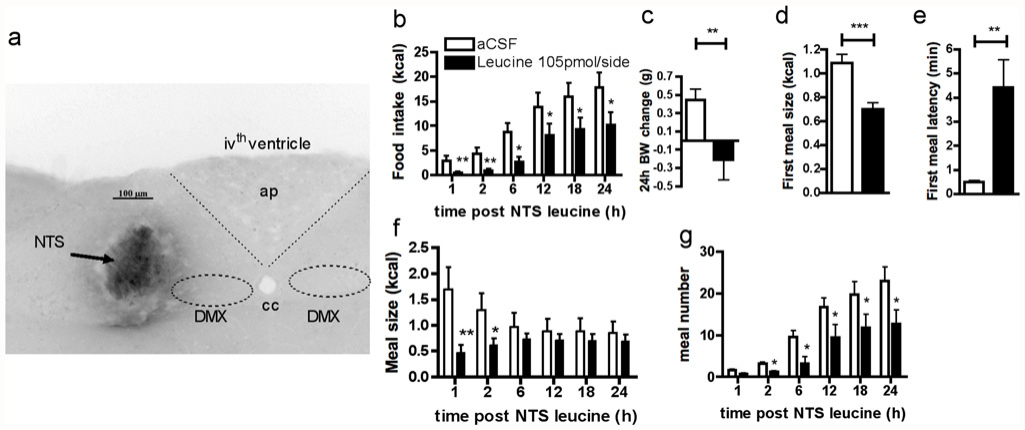
cmNTS L-leucine reduces food intake and body weight gain in chow fed mice. (a) cmNTS distribution of fluorescent-labeled L-leucine following a cmNTS targeted injection with inverted colors. ap: area postrema, cc: central canal, DMX: vagus nerve X. (b) Food intake, (c) 24h body weight change, (d) first meal size, (e) first meal latency, (f) meal size and (g) meal number 6h fasted mice following a cmNTS injection of L-leucine or vehicle. n = 10–12, *: P<0.05, **: P<0.01

Mice fasted for 6h received a 1 min NTS injection (50nl per side at 50 nl/min) of artificial cerebrospinal fluid (aCSF, Harvard Apparatus) alone or together with 2.1mM or 21mM L-leucine (Sigma Aldrich) 1 h before the onset of dark. Using fluorescently-labeled leucine (Phoenix Pharmaceuticals), we confirmed that these injections targeted the cmNTS, spreading throughout the rostrocaudal extent of the area postrema, from approximately-7.3 to-7.7 mm posterior to bregma (Paxinos and Franklin, 2001) (Fig. 1a).

#### Injection design 2

Mice were trained to rapidly eat a meal of their maintenance diet in the morning after an overnight fast. On the day of the test, overnight fasted mice were refed for 1h and then received a 1 min NTS injection (50 nl per side at 50 nl/min) of DMSO alone or together with 1.25 μg rapamycin (Calbiochem).

#### Injection design 3

Mice fasted for 6h received (1) a 1 min NTS injection (50 nl per side at 50 nl/min) of aCSF alone or together with 2.1mM or 21 mM L-leucine followed by (2) an ip injection of saline alone or with 1, 2 or 3 μg/kg BW non-sulfated CCK-8 (Bachem) 1 h before the onset of dark.

In all designs, access to food was restored immediately after the last injection. Food intake was continuously recorded for the following 24 h.

### Immunofluorescence

Mice were anesthetized with pentobarbital and received a transcardiac perfusion of 100ml of heparinized saline and 80 ml of 4% paraformaldehyde in KPBS. Brains were postfixed 48h in 30% sucrose 4% paraformaldehyde and sectioned using a freezing microtome. Free floating 30μm sections were blocked in 5% normal goat serum and incubated overnight at 4°C in phospho-S6 ribosomal protein (Ser235/236) rabbit antibody (1:50, Cell Signaling Technology), mouse anti-dopamine β hydroxylase (1:1000, Millipore) or mouse anti tyrosine hydroxylase (1:100, Immunostar) in 0.3% Triton X-100 and 5% NGS. Sections were then washed and incubated for 2 h with Alexa Fluor 488 or 596 secondary antibodies (Invitrogen).

### Micropunch dissection

Mice were decapitated and brains were quickly dissected and rinsed in ice-cold saline. The caudal brainstem section containing the NTS through the rostrocaudal extent of the area postrema (7.3–7.7 mm caudal to bregma) was sliced, immersed in liquid nitrogen for 2s, and the dorsovagal complex was rapidly punched out and immediately prepared for protein extraction.

### Western blot analysis

Brain micropunches were homogenized in 50mM Tris, 1 mM EGTA, 1 mM EDTA, 50 mM sodium fluoride, 10 mM β-glycerophosphate, 20 mM sodium pyrophosphate, 2 mM orthovanadate, 2mM PMSF, 1% Triton, and Complete phosphatase inhibitor cocktail (Roche). Protein extracts (10–20 **μ**g) were run on Criterion gels (Bio-Rad) and blotted onto nitrocellulose membranes. Immunoblots were incubated in primary antibodies against phospho-p70 S6 kinase (Thr389) or p70 S6 kinase (both from Cell Signaling Technologies), followed by HRP-linked secondary antibodies. Proteins were detected using enhanced chemiluminescence (ECL Plus, GE Healthcare). Band intensity was determined using NIH Image J software.

### Statistical analysis

All data, presented as means ± SEM, were analyzed using GraphPad Prism 5. For all statistical tests, an α risk of 5% was used. Multiple comparisons were tested with one-way or two-way ANOVAs for repeated measures and adjusted with Bonferroni post-tests. Single comparisons were made using one-tail Student *t* tests.

## Results

### cmNTS L-leucine administration reduces food intake in mice

We first assessed the behavioral and metabolic consequences of cmNTS L-leucine sensing in 10-week-old mice maintained on a regular chow diet. We used a dose of 105 pmol/side L-leucine (or 50nl per side of 2.1 mmol/L). This dose is consistent with the postprandial increase in hypothalamic parenchymal L-leucine concentration measured in rodents in response to the ingestion of a high-protein meal in microdialysis studies [18–20]. L-leucine in the CSF may be taken up by neurons via L-type amino acid transporter 1 (LAT1) [21] which is ubiquitously expressed in brain microvessels and circumventricular organs [22]. Administration of L-leucine into the cmNTS of 6h-fasted mice robustly reduced food intake and 24h body weight gain (Fig. 1b and 1c). The reduction of food intake occurred rapidly after the injection, as evidenced by the lower first meal size and first meal latency following the L-leucine injection compared to the vehicle injection (Fig. 1d and 1e). Lower food intake in L-leucine treated animals was maintained throughout the 24h following the injection, owing to a decrease in meal size for the first 2h following the injection (Fig. 1f), and a decrease in meal frequency starting 2h after the brain injection (Fig. 1g). Importantly, previous studies indicate that intracerebroventrivular L-leucine fails to induce the formation of conditioned taste aversion [23]. In vehicle-injected controls, the amount of calories ingested 1h to 24h after the cmNTS injection are in the range of previously reported values in mice [17; 24], supporting the conclusion that the injection procedure did not induce a non-specific aversive response to NTS or AP (area postrema) activation.

### HF feeding attenuates the anorectic effects of cmNTS L-Leucine

**Fig 2:**
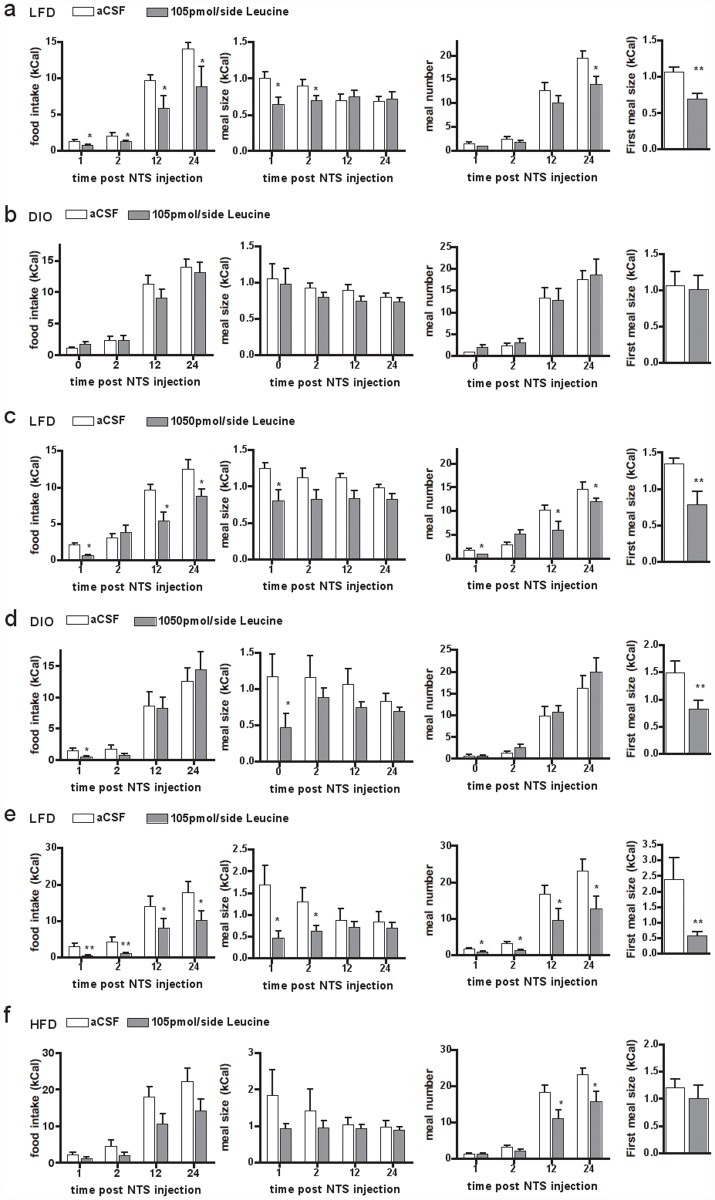
Impaired anorectic effect of cmNTs L-leucine in HF-fed and diet-induced obese mice. 24h food intake, meal size, meal number and first meal size following a cmNTS injection of aCSF or L-leucine 105pmol/side in mice fed a LF- or HF-diet for 6 months (a, b), 1050pmol/side in mice fed a LF- or HF-diet for 6 months (c,d), or 105pmol/side in mice fed a LF- or HF-diet for 2 weeks (e,f). N = 5–6, *: P<0.05. **: P<0.01

To test the consequences of DIO on cmNTS L-Leucine sensing, we measured the feeding response to cmNTS L-leucine administration in mice fed a HF diet or a LF control diet for 6 months from weaning, using a similar paradigm as that used in young mice. HF-fed mice consumed more calories over 24h (average 24h calorie intake over the 5 days preceding the first brain injection: 17.8±2.5 vs. 24.5±1.8 kCal, p = 0.02 in LF vs. HF-fed mice), were significantly heavier (body weight after surgical recovery: 30.68 ± 1.69 g vs. 38.45 ± 5.18 g in LFD vs DIO mice, p<0.001) and had an increased fat mass (fat mass after surgical recovery: 16.2 ± 1.6 g vs. 5.16 ± 0.5 g in LF vs DIO mice, p<0.001) compared to LF-fed controls. In age-matched LF-fed mice, bilateral cmNTS administration of 105 pmol per side L-leucine produced an anorectic response similar to that measured in young LF-fed mice (Fig. 2a). In contrast, the same dose of L-leucine failed to suppress food intake in DIO mice (Fig. 2b).

We then assessed the feeding response to a bilateral injection of a 10 times higher dose of L-leucine (1050 pmol/side) injected into the cmNTS in DIO mice. In age-matched LF-fed mice, bilateral cmNTS administration of 1050 pmol per side L-leucine produced an anorectic response similar to that measured in young LF-fed mice (Fig. 2c). In DIO mice, this dose of L-leucine acutely suppressed food intake, as evidenced by the reduced first meal size and 1h food intake (Fig. 2d), but 24h food intake and 24h body weight change remained the same as following vehicle administration (Fig. 2d). In addition, first meal latency (4.51±1.49 min vs. 5.65±2.77 min, p = 0.36 following aCSF or L-leucine treatment) and meal frequency (Fig. 2d) were not significantly altered following the 1050 pmol/side L-leucine injection into the cmNTS.

To determine whether the lack of response to cmNTS L-leucine measured in DIO mice was a consequence of their chronic exposure to HF feeding and increased adiposity, or a dysfunction occurring early during the development of DIO, we tested the feeding response to cmNTS L-leucine in mice fed a HF diet for 2 weeks beginning at 8 weeks of age. Two weeks of HF feeding did not significantly alter body weight or adiposity (starting body weight: 22.6 ± 0.5 vs. 22.6 ± 0.8 g, body weight after 2 weeks: 24.2± 0.6 vs. 25.2 ± 0.7 in LF vs HF-fed mice, p = 0.18, n = 6). In age-matched LF-fed mice, bilateral cmNTS administration of 105 pmol per side L-leucine produced an anorectic response similar to that measured in chow-fed mice (Fig. 2e). Injection of 105 pmol/side L-leucine into the cmNTS of mice fed a HF diet for 2 weeks did not reduce first meal size and did not suppress 1h and 2h food intake (Fig. 2f) and did not change first meal latency (data not shown). Long-term anorectic consequences of cmNTS leucine were attenuated in this group (differences at 12h and 24h not statistically different). However, the comparison of the feeding kinetics following aCSF or leucine treatment indicates that leucine produced a significant overall change in feeding in mice fed a HF-diet for 2 weeks (overall effect of the injection p = 0.038 in repeated measures 2-way ANOVA testing) (Fig. 2f). Meal pattern analysis indicate that decreased food intake following cmNTS L-leucine administration was a consequence of reduced meal frequency (Fig. 2f), with no change in meal size (Fig. 2f). Together, these data indicate that 2-week HF-feeding attenuates the acute anorectic consequences of cmNTS leucine administration.

### DIO impairs cmNTS mTORC1 /p70 S6 kinase 1 signaling

**Fig 3:**
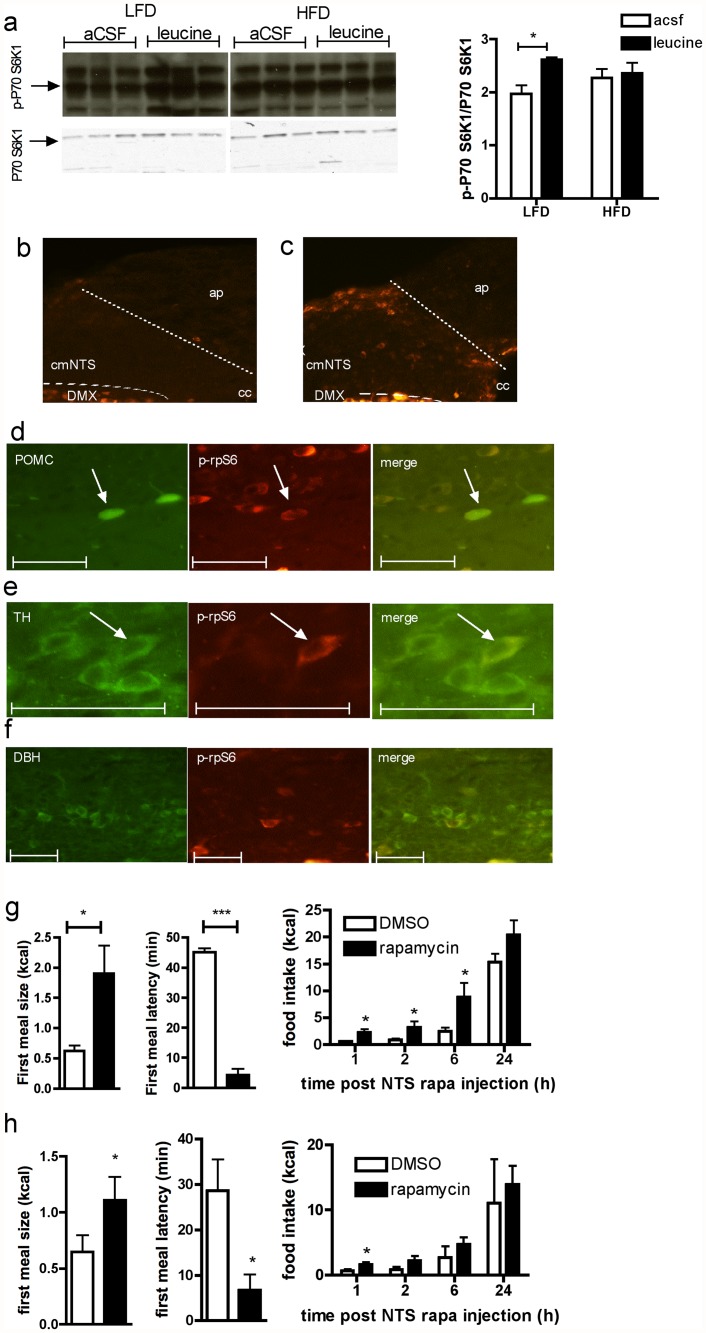
Impaired activity of the cmNTS p70 S6 kinase 1 pathway in HF-fed mice. p70 S6 kinase 1 Thr389 phosphorylation in the cmNTS of (a) chow-fed mice or HF-fed mice (n = 5–6). Distribution of phosphorylated S6 ribosomal protein in the cmNTS of 6h-fasted (b) chow-fed mice or (c) HF diet-fed mice. Colocalization (right, orange) or phosphorylated ribosomal protein S6 (p-rpS6, middle, red) and POMC (d), TH (e) or DBH (f) (left, green) in the cmNTS of 6h-fasted HF-fed mice. Scale bar: 50 μm. First meal size, first meal latency and food intake in LF-fed (g, n = 5) or HF-fed (h, n = 5) pre-sated mice following a cmNTS injected of rapamycin or vehicle. *: P<0.05.

Activation of the amino acid sensing mTORC1/p70 S6 kinase 1 pathway following central intracerebroventricular or hypothalamic parenchymal L-leucine administration has been previously implicated in the anorectic effect of central L-leucine [17; 23], as well as in the anorectic effect of cmNTS L-leucine in rats [8]. To confirm that this pathway is also responsive to local cmNTS L-leucine injection in mice, we measured the expression of the activated phosphorylated form of p70 S6 kinase 1, a downstream effector of mTORC1, in dorsovagal complex (DVC) micropunches collected 30 min after a cmNTS injection of a feeding inhibitory dose of L-leucine or aCSF. cmNTS L-leucine injection significantly increased p70 S6 kinase 1 phosphorylation compared to aCSF injection (Fig. 3a) in mice fed a LFD. In contrast, in mice fed a HF diet for 2 weeks, cmNTS L-leucine failed to increase DVC P70 S6 kinase 1 phosphorylation (Fig. 3a). Interestingly, these blots (all samples were processed together on the same blot) indicate that after aCSF administration, P70 S6 kinase 1 activity tended to be higher in HF-fed mice than in LFD controls (P = 0.08), suggesting that baseline activity of the mTORC1/p70 S6 kinase 1 pathway is increased in the cmNTS following 2 weeks of HF feeding. This was confirmed by evaluating basal levels of phosphorylated ribosomal protein S6 (p-rpS6), a downstream effector of mTORC1 and p70 S6 kinase 1 in fasted uninjected animals. In fasted LF-fed controls, p-rpS6 was absent from the cmNTS (Fig. 3b), whereas p-rpS6 was expressed in the cmNTS of fasted HF-fed mice (Fig. 3c). Immunofluorescent colocalization of p-rpS6 with POMC (using POMC-GFP mice), tyrosine hydroxylase (TH) or dopamine beta-hydroxylase (DBH) in cmNTS brain sections of fasted HF-fed mice indicated that some cmNTS cells expressing p-rpS6 also expressed POMC (Fig. 3d) or TH (Fig. 3e). However, we failed to observe colocalization of DBH with prpS6 (Fig. 3f).

### Inhibition of cmNTS mTORC1 signaling promotes feeding in DIO mice

We previously found that cmNTS application of the mTORC1 inhibitor rapamycin promotes feeding in chow fed rats [8]. To test whether the orexigenic response to mTORC1 inhibition is preserved in DIO mice, we measured the feeding consequences of local cmNTS rapamycin injection in presated DIO mice or LF-fed controls. In mice maintained on LF, rapamycin rapidly induced feeding, as evidenced by a significant decrease in first meal latency and a significant increase in first meal size (Fig. 3g). Increased food intake in rapamycin treated mice was maintained for 6h following the injection (Fig. 3g) due to an increase in meal size, with no change in meal frequency (data not shown). In DIO mice, the orexigenic response to rapamycin was maintained, albeit at a reduced level. First meal size was significantly increased and first meal latency was significantly decreased following cmNTS rapamycin administration compared to vehicle (Fig. 3h). However, the rapamycin-induced hyperphagia was not sustained in DIO mice, and cumulative food intakes were not significantly different following rapamycin and vehicle treatment beginning 2h after the brain injections.

### HF feeding impairs cmNTS integration of meal-related signals

**Fig 4:**
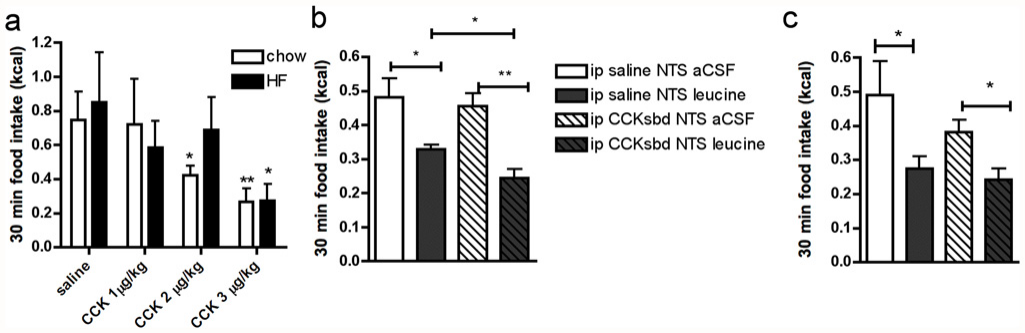
Impaired cmNTS metabolic integration in DIO mice. (a) 30 min food intake in chow-fed or HF-fed mice following ip injection of different doses of CCK. 30 min food intake following the combination of an anorectic dose of L-leucine injected into the cmNTS together with a subthreshold ip CCK dose in (b) chow-fed mice (leucine: 105pmol/side, CCK: 1 **μ**g/kg) or (c) high-fat fed (leucine: 1.05nmol/side, CCK: 2 **μ**g/kg) mice. n = 5–6, *: P<0.05, **: P<0.01

Our previous work supports the idea that the cmNTS integrates information arising from circulating nutrients and gut-derived signals to regulate meal size [8]. To test the hypothesis that DIO impairs the ability of the cmNTS to integrate signals arising from circulating nutrients with the gut satiety peptide CCK, we first identified a subthreshold dose of ip CCK that had no significant anorectic effect in mice fed a LF or HF-diet for 6 months (Fig. 4a). We then combined a cmNTS injection of an anorectic dose of L-leucine (105 pmol/side in LF- fed mice, 1.05nmol/side in HF-fed mice) or vehicle with a subthreshold dose of ip CCK (1 μg/kg in controls, and 2 μg/kg in HF-fed mice) or vehicle. In controls, we confirmed that the combination of ip CCK and cmNTS leucine produced a synergistic suppression of food intake (Fig. 4b). Mice receiving both the ip injection of CCK and the cmNTS injection of L-leucine had a 25% further reduction in food intake compared to mice that received L-leucine alone. In contrast, a subthreshold dose of ip CCK did not modify the reduction of food intake induced by an anorectic dose of cmNTS L-leucine in HF-fed mice (Fig. 4c).

## Discussion

Hyperphagic obesity is characterized by an increase in meal size that contributes to increased daily energy intake [11]. The cmNTS is a key region implicated in the regulation of meal size through its processing of post-ingestive neural inputs arising from vagal afferents and descending cortico-limbic inputs, as well as inputs deriving from the local brainstem detection of circulating nutritional and hormonal signals of fuel availability. As little is known about the consequences of HF feeding on cmNTS nutrient sensing and integrative functions in the control of feeding, we characterized the behavioral and metabolic consequences of cmNTS L-leucine detection in HF-fed and DIO mice, we investigated the consequences of HF feeding on cmNTS metabolic integration, and we identified mechanisms implicated in the changes in cmNTS nutrient sensing in HF-fed non-obese and obese mice.

Our results extend our previous report [8] supporting a role for the cmNTS as a nutrient-sensing region engaged in the feedback inhibitory regulation of feeding and the integration of multiple metabolic signals, and indicate that this regulation is conserved in mice and rats. Our results also identify in mice maintained on a LF diet that cmNTS L-leucine sensing reduces feeding through multiple behavioral changes, both acutely through an increase in meal latency and a decrease in meal size, and over the longer term through a decrease in meal number.

Here we show for the first time that DIO in mice is associated with a desensitization to the anorectic effect of local cmNTS L-leucine administration. Interestingly, increasing the leucine dose restored the ability of leucine to reduce acute food intake in HF-fed DIO mice, but failed to restore the longer term suppression of feeding across the ensuing 24 hours. In contrast, in non-obese mice fed a HF diet for only 2 weeks, cmNTS L-leucine failed to acutely suppress feeding but significantly reduced 24h food intake. These observations demonstrate that the acute feeding-suppressive effects of cmNTS L-leucine administration are impaired early during the development of DIO, independently of adiposity, and suggest that the impairment in the long term feeding suppressive effects of cmNTS L-leucine is not a primary consequence of high fat feeding, but instead is related to increased adiposity, possibly via elevated leptin levels.

To further characterize the consequences of short term high fat feeding on the acute regulation of meal size in response to acute cmNTS nutrient application, we assessed the caudal brainstem DVC activity of the amino-acid sensing mTORC1 pathway. Our results suggest a role for constitutive activation of cmNTS mTORC1 signaling in the lack of acute feeding response to cmNTS L-leucine in HF-fed mice. This conclusion is supported by the higher baseline activity level of the mTORC1 effector p70 S6 kinase 1 in the cmNTS of HF-fed mice compared to chow fed controls. Increased baseline mTORC1 activity as a consequence of HF feeding has been consistently reported in several tissues including the liver, adipose tissue, muscle and hippocampus [25–27], but this result contrasts with a study reporting that HF feeding reduced mTORC1 signaling in the hypothalamus [28]. Interestingly, several reports indicate that the activity of nutrient-sensing signaling pathways implicated in the central control of energy metabolism, such as the AMPK or Sirt1 signaling pathways, is altered in response to HF feeding and DIO [29–31]. The lack of L-leucine—induced increase in p70 S6 kinase 1 activity in mice maintained on HF diet for 2 weeks provides further evidence in favor of impaired cmNTS mTORC1 signaling as a mechanism underlying the lack of acute anorectic response to cmNTS L-leucine. Thus, we speculate that hyperphagia in HF-fed mice is at least in part mediated by a chronic elevation in cmNTS mTORC1 activity that reduces the dynamic range of mTOR activation in these neurons, effectively making them less sensitive to normal feedback stimulation. Consistent with this hypothesis, the orexigenic response to pharmacological cmNTS mTORC1 inhibition is preserved in HF-fed mice. Thus, reduction in cmNTS mTORC1 tone, through rapamycin treatment or caloric restriction, might be predicted to restore the dynamic range of cmNTS leucine-induced mTORC1 signaling important in the negative feedback the control of ingestion.

The fact that cmNTS rapamycin was able to rapidly stimulate feeding in both LF and DIO animals suggests: 1) a strong constitutive feeding inhibitory circuitry operating at the level of the cmNTS, and 2) that acute mTORC1 inhibition in the cmNTS produces an acute change in local neuronal polarization state and a consequent alteration in feeding modulatory peptide signaling. These suggestions raise the important mechanistic questions of which NTS neurotransmitter and/or neuropeptide signals may keep feeding at bay. Further studies are needed to confirm the role of melanocortinergic and catecholaminergic circuits in the ability of brainstem rapamycin to regulate meal size.

Last, we examined the short term feeding consequences of combinations of local nutrient infusions of leucine and peripheral administration of the gut satiety peptide CCK. We found, as in rats, that this combination of nutrient stimulation and gut satiety peptide produces a greater inhibition of acute food intake and meal size than either stimulus alone. However, in DIO mice, the combination of a subthreshold dose of ip CCK with an anorectic dose of L-leucine failed to produce a synergistic suppression of food intake. These data demonstrate that cmNTS mechanisms of metabolic integration are impaired in DIO.

Thus, this study identified two novel mechanisms through which the post-ingestive regulation of meal size is impaired in HF feeding: 1) a desensitization to the anorectic effect of amino acid nutrient stimuli at the level of the cmNTS, and 2) an impairment in the mechanisms through which various metabolic signals synergistically interact to control feeding. The intracellular signaling cascades mediating synergistic feeding inhibitory interactions between brainstem nutrient and gut peptide stimuli, and how they are altered during DIO, remain to be determined. Future characterization of these mechanisms will likely provide novel targets for effective combination therapies in the treatment of obesity.
